# Troyka represents a unique lineage of virus-like retroelements

**DOI:** 10.1099/jgv.0.002143

**Published:** 2025-09-15

**Authors:** Kenji K. Kojima

**Affiliations:** 1 Genetic Information Research Institute, East Palo Alto, CA 94303, USA

**Keywords:** *Gypsy*, LTR retrotransposon, *Metaviridae*, revtraviricetes, *Troyka*

## Abstract

Eukaryotic reverse transcriptase genes are mostly incorporated into viruses or transposons. Among the six reported families of reverse-transcribing viruses, three families (*Metaviridae*/*Gypsy*, *Belpaoviridae*/*BEL* and *Pseudoviridae*/*Copia*) have proliferated mostly as transposons, collectively known as LTR retrotransposons. *Troyka* was reported as a unique lineage of the *Metaviridae*/*Gypsy* family. While most LTR retrotransposons generate 4 to 6 bp target site duplications (TSDs) upon integration and contain 5′-TG. CA-3′ termini, *Troyka* generates 3 bp TSDs and contains 5′-CG.CG-3′ termini. Here, the distribution and diversity of *Troyka* were extensively investigated from the available genome sequences. In addition to the six animal phyla reported previously, *Troyka* was characterized for the first time in Hemichordata, Priapulida, Annelida, Phoronida and Brachiopoda. The unique terminal nucleotides of *Troyka* are very well conserved. The phylogenetic analysis with various combinations of conserved domains supported the independent position of *Troyka* from any established retroelement groups, most likely as the sister lineage of the group including *Metaviridae*/*Gypsy*, *Retroviridae*, *Caulimoviridae*, Lokiretroviruses and *Odin* LTR retrotransposons. *Troyka* is here proposed as a new superfamily of LTR retrotransposons.

**Impact Statement**

This article introduces a new superfamily of LTR retrotransposons, showing the unseen diversity of virus-like retroelements. The reported information about the unique characteristics of *Troyka* LTR retrotransposons would encourage the improvement of currently available tools for repeat annotation, thus contributing to the better understanding of genome organization and evolution.

## Data Availability

Supplementary material is available with the online version of this article, available through Figshare at https://doi.org/10.6084/m9.figshare.29341544 [1]

## Introduction

Reverse transcriptase (RT) is an enzyme that catalyses DNA polymerization using an RNA template [2,3]. In eukaryotes, except for two cellular genes, telomerase reverse transcriptase (TERT) and reverse transcriptase (RVT), RT genes are seen in two forms of selfish DNA or RNA entities: transposons (retrotransposons) and viruses [4,5]. Phylogenetic analysis divided the RT into four major branches [4]: prokaryotic retroelements (P), non-LTR retrotransposons and RVT (L), *Penelope*-like elements and TERT (T), and other eukaryotic retroelements, including all viral forms of retroelements (V).

Reverse-transcribing viruses are classified into six families in the class *Revtraviricetes* by the International Committee on Taxonomy of Viruses (https://ictv.global/taxonomy). They are *Retroviridae* [6], *Caulimoviridae* [7], *Hepadnaviridae* [8], *Pseudoviridae* [9], *Belpaoviridae* [10] and *Metaviridae* [11,12]. Among them, the former three groups are full-fledged viruses, which can transmit between cells and whose virus particles are observed. However, it is considered that most elements in the latter three families are more likely to behave as a transposon, and thus, they are also called *Copia* (or *Ty1/Copia*), *BEL* (or *BEL/Pao*) and *Gypsy* (or *Ty3/Gypsy*) superfamilies of LTR retrotransposons, respectively [13,14]. It is notable that ‘family’ in the virus taxonomy almost corresponds to ‘superfamily’ in the transposon classification. These LTR retrotransposons generally encode one (gag-pol) or two proteins (gag and pol). Gag is a structural protein that shows similarity to the capsid and nucleocapsid proteins of vertebrate retroviruses (*Retroviridae*) [15]. Pol contains several enzymatic domains: retropepsin (RP or protease), RT, ribonuclease H (RH) and DDD/E transposase/integrase (IN). In *Retroviridae*, the original RH has lost the enzymatic function to become the ‘tether domain’ and a new RH has been acquired [16]. Some LTR retrotransposons encode additional proteins or protein domains. Chromovirus is a group of *Gypsy* LTR retrotransposons that acquired a chromodomain at the C-terminus of pol protein [14,17]. Several independent groups of LTR retrotransposons have acquired an envelope glycoprotein gene from viruses; Errantivirus has acquired an envelope gene likely from baculovirus [18], and *Anakin* has acquired an envelope gene from chuvirus [19]. Oppositely, not a few retrovirus lineages have lost their envelope gene secondarily and have become a ‘retrotransposon’; these retroviruses are called ‘endogenous retroviruses (ERVs)’ [20]. *Odin* LTR retrotransposons found in Cnidarians constitute a sister lineage of Lokiretroviruses and might be another example of endogenous retroviruses [21].

Phylogenetic analyses revealed the monophyly of the six families of reverse-transcribing viruses along with several non-viral and viral groups, but distinct from the two other major groups of eukaryotic retrotransposons: non-LTR retrotransposons and *Penelope*-like elements [4]. The retroelement groups which show close affinity to the reverse-transcribing viruses are HEART, Nackednavirus and *DIRS*. HEART is an enigmatic group of retroelements whose mobilization mechanism is not yet resolved [22]. Nackednavirus represents a sister lineage of *Hepadnaviridae* but lacks an envelope protein [23]. *DIRS*, which may be further classified into *PAT*, *Ngaro*, *VIPER* and *TATE*, are a unique group of retrotransposons whose RT is closely related to that of LTR retrotransposons but which encodes a tyrosine recombinase instead of DDD/E transposase for its genome integration [24,26]. Although the phylogenetic relationships among these retroelements have not yet been confirmed, the monophyly of *Retroviridae*, *Caulimoviridae* and *Metaviridae*/*Gypsy* was indicated based on the RT phylogeny [16,21, 27]. It is consistent with the conserved domain order in the Pol proteins of retroviruses and *Metaviridae*/*Gypsy*, as well as of *Belpaoviridae*/*BEL* [14].

*Troyka* is a small group of LTR retrotransposons that are assigned to the *Gypsy* superfamily in the current Repbase taxonomy. *Troyka* was first reported from the cnidarian *Nematostella vectensis* and the amphioxus *Branchiostoma floridae* in 2008 [28]. To date, *Troyka* has been reported from six animal phyla, Cnidaria, Chordata, Echinodermata, Arthropoda, Nemertea and Mollusca [13]. *Troyka* is unique in its terminal signatures: 5′-CG.CG-3′, while most LTR retrotransposons terminate with 5′-TG and CA-3′. *Troyka* generates 3 bp target site duplications (TSDs), while other LTR retrotransposons generate 4 to 6 bp TSDs. These unique sequence features led me to reinvestigate the position of *Troyka* in the RT phylogeny.

In this study, the wide distribution, terminal signature and the 3 bp TSDs of *Troyka* LTR retrotransposons are confirmed. The phylogenetic analysis of virus-like retroelements revealed that *Troyka* constitutes an independent branch from any reported virus-like retroelement groups.

## Methods

### Characterization of *Troyka* LTR retrotransposons

All *Troyka* LTR retrotransposon sequences were downloaded from Repbase (https://www.girinst.org/repbase/) [13] as of 24 July 2024.

blast search was performed with reported *Troyka* proteins in Repbase as queries against protein datasets or genome DNA sequence datasets at the National Center for Biotechnology Information (NCBI) blast website (https://blast.ncbi.nlm.nih.gov/Blast.cgi). Genomes with significant blast hits were downloaded from the NCBI Assembly (https://www.ncbi.nlm.nih.gov/datasets/genome/). As many significant blast hits resulted in the characterization of non-*Troyka* retroelements, such as *Metaviridae/Gypsy*, only the genome assemblies from which *Troyka* elements were characterized in this study are listed in Table S1, available in the online Supplementary Material.

Censor [29] searches were performed with the protein of *Troyka-1_TeGr* from the blood clam *Tegillarca granosa* in Repbase as queries against each genome assembly. Censor hits were extracted and clustered with BLASTCLUST 2.2.25 in the NCBI blast package with the thresholds at 75% length coverage and 75% sequence identity. The consensus sequence for each cluster was generated with the 50% majority rule applied with the help of homemade scripts. Censor searches were again performed with the consensus sequence of each cluster against the respective genome assembly. Up to 10 Censor hits were extracted with 10,000 bp flanking sequences on both sides. Consensus sequences were regenerated to be elongated to find LTRs. The full-length consensus sequences were determined based on the presence of LTRs at both ends and flanking 3 to 6 bp TSDs. All characterized consensus sequences are available as supplementary materials (Data S1) and are also submitted to Repbase (https://www.girinst.org/repbase/).

### Terminal signatures and TSDs

To confirm the termini and TSDs, four groups of organisms whose genomes have been sequenced are chosen for the analysis: four *Polites* species (*Polites rus*, *Polites lutea*, *Polites cylindrica* and *Polites divaricata*) in Cnidaria, five *Mytilus* species (*Mytilus galloprovincialis*, *Mytilus californianus*, *Mytilus coruscus*, *Mytilus edulis* and *Mytilus trossulus*) in Mollusca, three *Heliocidaris* species (*Heliocidaris crassispina*, *Heliocidaris erythrogramma* and *Heliocidaris tuberculata*) in Echinodermata and five species in Leuciscinae (*Rutilus rutilus*, *Abramis brama*, *Squalius cephalus*, *Scardinius erythrophthalmus* and *Vimba vimba*) in Chordata. Censor searches were performed with the genomes of *P. rus*, *M. trossulus*, *H. erythrogramma* and *R. rutilus* against the *Troyka* consensus sequences from these respective genomes. The entire *Troyka* sequence and flanking sequences were extracted. The junctions and TSD were determined manually. The entire *Troyka* and one TSD sequence are removed to prepare the pre-inserted sequence. Censor searches were performed with the genomes of sibling species against the pre-inserted sequence to detect the orthologous loci.

### Sequence alignment and phylogenetic analysis

The protein sequences longer than 700 residues encoded by *Troyka* LTR retrotransposons were aligned with MAFFT [30] with default parameters. The alignment was manually investigated to remove weakly aligned sequences, caused by frameshift or deletion. The final alignment contains 457 sequences of 1,409 sites. A maximum likelihood tree was generated at the PhyML 3.0 server (http://www.atgc-montpellier.fr/phyml/) [31] with an approximate likelihood-ratio test. The substitution model Q.insect+R+F was used based on the Bayesian information criterion (BIC). The phylogenetic tree was rooted at the midpoint and visualized with FigTree v.1.4.3 (http://tree.bio.ed.ac.uk/software/figtree/).

To examine the relationship of *Troyka* to other groups of virus-like retroelements, the alignments of protein domains from *Metaviridae*/*Gypsy*, *Belpaoviridae*/*BEL*, *Pseudoviridae*/*Copia*, *Retroviridae* and *Caulimoviridae* were downloaded from the Gypsy Database (https://gydb.org/) [14] on 24 July 2024. *DIRS*, *ERV4* [32] and endogenous foamy virus [33] sequences were extracted from Repbase (https://www.girinst.org/repbase/) [13] and selected to represent the diversity of these groups. Nackednaviruses [23], HEART [22], Pliego virus [34], Lokiretroviruses [35] and *Odin* LTR retrotransposons [21] were obtained from the sequences reported in publications.

The multiple alignment of each domain (RP, RT, RH and IN) was generated using MAFFT [30] and muscle [36] with default parameters. As well as the single domain alignments, the concatenated sequences of multiple domains were also used for the phylogenetic analysis. The alignments used for the phylogenetic analysis were (1) the RT domain only, composed of 370 sequences of 573 sites; (2) the concatenated sequences of RT and RH domains, composed of 298 sequences of 875 sites; (3) the concatenated sequences of RP, RT and IN domains, composed of 243 sequences of 1,238 sites; and (4) the concatenated sequences of RP, RT, RH and IN domains, composed of 173 sequences of 1,336 sites.

Maximum likelihood trees were generated at the PhyML 3.0 server (http://www.atgc-montpellier.fr/phyml/) [37] with 100 bootstrap supports. The substitution model Q.pfam+R+F was selected based on the BIC. The phylogenetic tree was unrooted and visualized with FigTree v.1.4.3 (http://tree.bio.ed.ac.uk/software/figtree/).

## Results and discussion

### Phylogenetic distribution

Repbase keeps LTR retrotransposons and retroviruses split into two parts: LTR and internal portion. As of 24 July 2024, Repbase [13] contained 34 *Troyka* entries (17 LTRs, 16 complete internal portions and 1 incomplete internal portion) from 11 species of 6 animal phyla (Cnidaria, Arthropoda, Mollusca, Nemertea, Echinodermata and Chordata). Although the analysis was not systematic, this study characterized 586 LTRs and 589 internal portions from 256 species (Tables 1 and S1). Among them, 581 pairs of LTRs and internal portions were annotated. The internal portions for the five LTRs were not determined. The LTRs of the three internal portions were completely identical to the LTRs of sibling species. The LTR for *Troyka-1_MagHon-I* from *Magallana* (*Crassostrea*) *hongkongensis* is identical to *Troyka-2_MagAng-LTR* from *Magallana angulata*, the LTR for *Troyka-1_OxySte-I* from *Oxygymnocypris stewartii* is identical to *Troyka-1_GymEck-LTR* from *Gymnocypris eckloni* and the LTR for *Troyka-3_EptAta-I* from *Eptatretus atami* is identical to *Troyka-3_EptOki-LTR* from *Eptatretus okinoseanus*. They represent the activity of *Troyka* in the common ancestors of sibling species. The remaining five internal portions correspond to internally deleted derivatives.

**Table 1.** Distribution of *Troyka* LTR retrotransposons. The numbers of species whose genomes contain *Troyka* sequences are shown in parentheses

Phylum	Class	Order (no. of species with *Troyka*)
Cnidaria	Hexacorallia	Actiniaria (6), Scleractinia (17), Antipatharia (1)
	Octocorallia	Alcyonacea (1), Scleralcyonacea (2), Malacalcyonacea (1)
	Hydrozoa	Leptothecata (1), Anthoathecata (2), Siphonophorae (1)
	Scyphozoa	Semaostomeae (2), Rhizostomeae (2)
	Cubozoa	Carybdeida (1)
Echinodermata	Crinoidea	Comatulida (2)
	Asteroidea	Forcipulatida (5), Valvatida (2), Paxillosida (2)
	Ophiuroidea	Amphilepidida (3)
	Echinoidea	Temnopleuroida (2), Camarodonta (5), Clypeasteroida (1), Diadematoida (2)
	Holothuroidea	Aspidochirotida (1), Apodida (1)
Hemichordata	Enteropneusta	Harrimaniidae^*^ (1), Ptychoderidae* (1)
Chordata	Leptocardii	Amphioxiformes (3)
	Ascidiacea	Stolidobranchia (2)
	Myxini	Myxiniformes (4)
	Chondrichthyes	Heterodontiformes (1)
	Actinopterygii	Notacanthiformes (1), Anguilliformes (2), Clupeiformes (10), Cypriniformes (38), Characiformes (1), Stomiiformes (1), Myctophiformes (4), Lampriformes (2), Gadiformes (2), Kurtiformes (1), Blenniiformes (1), Carangiformes (2), Perciformes (5), Spariformes (2)
Arthropoda	Thecostraca	Balanomorpha (2), Scalpellomorpha (1), Sacculinidae^*^ (1)
	Malacostraca	Decapoda (2)
Nemertea	Pilidiophora	Heteronemertea (2)
Priapulida	Priapulimorpha	Priapulimorphida (1)
Mollusca	Bivalvia	Venerida (12), Adapedonta (2), Cardiida (9), Lucinida (7), Myida (4), Pterioida (4), Mytilida (11), Ostreida (13), Arcoida (3), Pectinida (5)
	Gastrapoda	Patellidae^*^ (4), Lottiidae* (2), Trochida (3), Lepetellida (4), Neomphalida (1)
	Cephalopoda	Seppida (2), Myopsida (2)
	Scaphopoda	Gadilida (1), Dentaliida (1)
	Polyplacophora	Chitonida (2)
	Caudofoveata	Chaetodermatida (1)
Annelida	Sipuncula	Golfingiida (1)
	Polychaeta	Xenopneusta (1), Phyllodocida (2), Eunicida (1), Sabellida (5), Scolecida (1), Spionida (1), Terebellida (2), Magelonidae^*^ (1)
Phoronida	–	Phoronidae^*^ (1)
Brachiopoda	Lingulata	Lingulida (1)

*No order is assigned.

In addition to the six animal phyla mentioned above, Hemichordata, Priapulida, Annelida, Phoronida and Branchiopoda also include species whose genomes have *Troyka* insertions. No *Troyka* was found outside of Metazoa.

*Troyka* is particularly widely distributed in Cnidaria and Mollusca. In Cnidaria, *Troyka* is distributed in five classes (Hexacorallia, Octacorallia, Hydrozoa, Scyphozoa and Cubozoa). Among the 30 Hexacorallia genomes analysed, 24 genomes contain at least 1 *Troyka* element. In Mollusca, *Troyka* is distributed in seven classes (Bivalvia, Gastropoda, Cephalopoda, Scaphopoda, Polyplacophora and Caudofoveata) and is especially rich in Bivalvia; among the 86 genomes analysed, 70 contain at least one *Troyka* element. Although the distribution is patchy in Chordata, out of 66 genomes from Cypriniformes, 38 contain *Troyka* insertions. In Arthropoda, *Troyka* was found only in two groups of crustaceans, Thecostraca (barnacles and crabhook barnacles) and Decapoda (shrimps). Although no genomic sequence of *Troyka* was characterized, the blast search against transcriptome shotgun assembly (TSA) in NCBI (https://blast.ncbi.nlm.nih.gov/Blast.cgi) suggested the presence of *Troyka* in the family Gammaridae in Amphipoda too (*Eulimnogrammarus verrucosus*, *Marinogammarus marinus*, *Gammarus lacustris* and *Hirondellea gigas*).

There is no instance of *Troyka* in terrestrial animals. *Troyka* was not observed from terrestrial Chordata (Amphibia, Reptilia, Aves and Mammalia), Arthropoda (Hexapoda, Myriapoda and Arachnida), Annelida (Clitellata) or Mollusca (Caenogastropoda and Heterobranchia in Gastropoda), despite the vast genome sequence information of mammals, birds and insects (Table 1). It suggests that *Troyka* has not successfully inhabited the land.

### Protein domain structure

The LTR length ranges between 100 and 400 bp. The internal portion is between 1,553 and 7,691 bp in length. The majority of *Troyka* LTR retrotransposons encode a single Gag-Pol protein which includes CCHC zinc finger, RP, RT, RH and IN, in the identical order of *Metaviridae*/*Gypsy* and *Belpaoviridae*/*BEL*. Only a few *Troyka* elements encode Gag and Pol as separate proteins. No *Troyka* element encodes an envelope protein. The HHpred [38] analysis with *Troyka* Gag proteins showed their similarity to the Gag proteins from *Copia*, *Ty3*, HIV-1 and the Gag-derived protein dArc [39] in both capsid and nucleocapsid domains. The zinc finger at the C-terminus of *Troyka* Gag protein can be shown as CX_2_CX_8-10_HX_4_C, which is distinct from CX_2_CX_4_HX_4_C seen in other virus-like retroelements (*Retroviridae*, *Metaviridae*/*Gypsy*, *Pseudoviridae/Copia*, *Belpaoviridae*/*BEL* and *Caulimoviridae*) [15]. No tether domain is present between RT and RH domains, unlike *Retroviridae*, Lokiretroviruses and *Odin* LTR retrotransposons. The GPF/Y motif, which is a hallmark of IN of *Metaviridae*/*Gypsy*, *Retroviridae*, *Odin* and *Ginger1* DNA transposons [40], was not found in *Troyka*, either.

Some short *Troyka* elements appear not to encode a functional Gag-Pol or Pol protein. Such non-autonomous elements are especially abundant in sea urchins (Echinoidea); 62 of 70 non-autonomous *Troyka* elements found in this study were from sea urchins.

### Transmission patterns

**Fig. 1.**
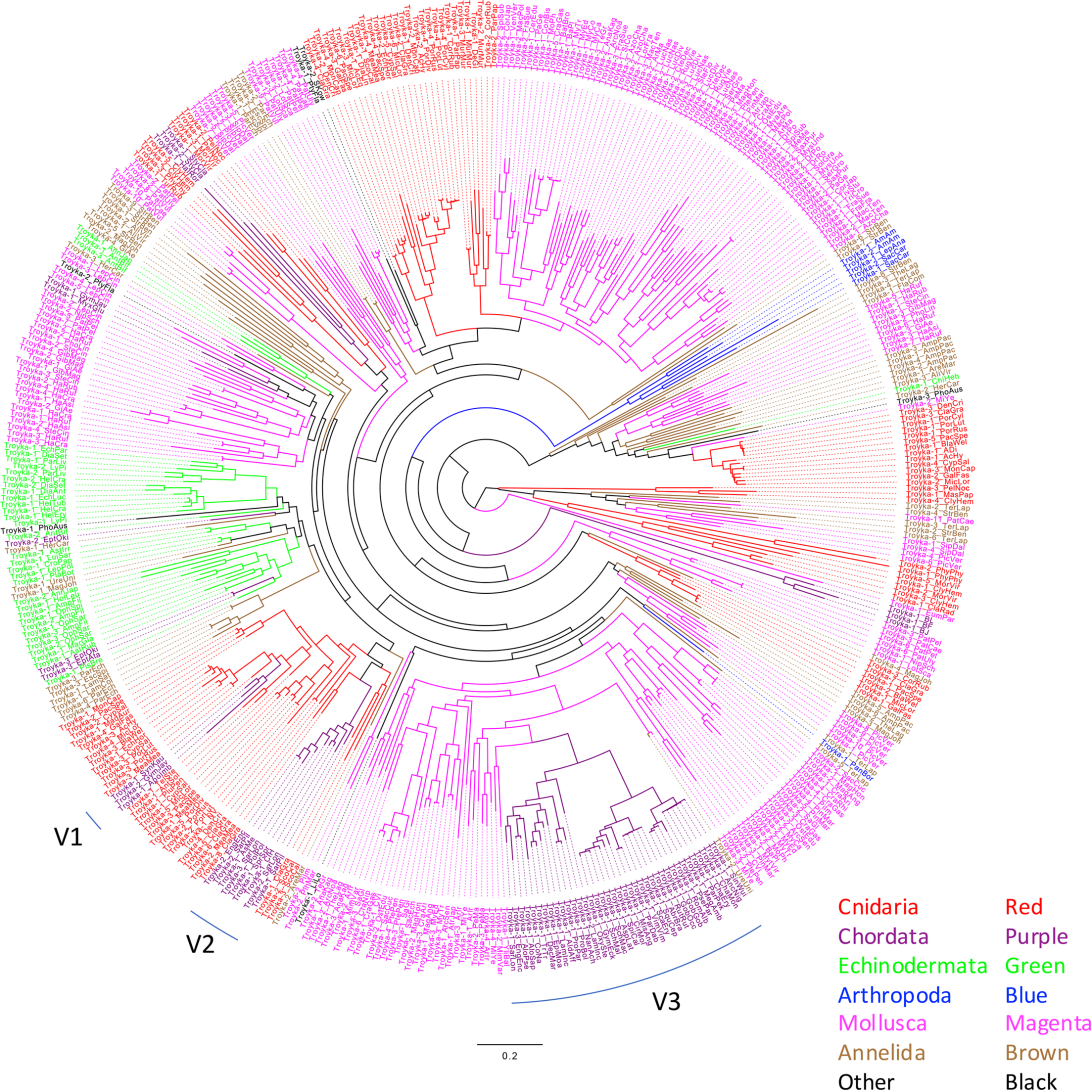
Phylogenetic tree of *Troyka* LTR retrotransposons based on the entire Pol protein. Branches are coloured based on the host phylogeny of *Troyka*. Three lineages from ray-finned fishes are indicated with arcs V1–V3. The original phylogenetic trees and protein alignments are available as Data S2 and S3. The species abbreviations in *Troyka* names are shown in Table S1.

The patchy distribution of *Troyka* in animals implies the horizontal transfer during evolution. The phylogenetic analysis using the entire *Troyka* Pol protein sequences revealed that *Troyka* elements from related organisms tend to cluster together, indicating their long-term vertical transmission (Fig. 1). At the same time, multiple events of horizontal transfer of *Troyka* were also indicated.

*Troyka* from ray-finned fishes constitutes three lineages. The lineage V1 consists of three *Troyka* elements from Anguilliformes (*Troyka-1_SynKau*, *Troyka-2_GymJav*) and Kurtiformes (*Troyka-1_ApoImb*). The lineage V2 consists of nine *Troyka* elements from Clupeiformes (*Troyka-1_ClH*, *Troyka-1_SprSpr*, *Troyka-1_SarPil*, *Troyka-2_EngEnc*, *Troyka-2_SarPil*, *Troyka-2_SalLon* and *Troyka-3_SarLon*), Characiformes (*Troyka-1_AsMe*) and Gadiformes (*Troyka-1_PolPol*). All other 38 *Troyka* elements from ray-finned fishes belong to the lineage V3, which includes *Troyka* from Cypriniformes, Clupeiformes, Carangiformes, Lampriformes, Notacanthiformes and Myctophiformes. The two fish genomes from Clupeiformes, European anchovy *Engraulis encrasicolus* and Indian oil sardine *Sardinella longiceps,* contain both V2 and V3 lineages of *Troyka*.

*Troyka* elements from the same family in the order Cypriniformes clustered together (Figs 1 and S1), suggesting their vertical inheritance. However, the relationships between host families and the *Troyka* phylogeny are not consistent (Fig. S1). The family Cobitidae belongs to the suborder Cobitoidei, while Xenocyprinidae, Gobionidae, Leuciscidae and Cyprinidae belong to the suborder Cyprinoidei [41]. *Troyka* elements from Cyprinidae are more closely related to those from Cobitidae than Lauciscidae, Gobionidae or Xenocyprididae. This discrepancy could be explained by the horizontal transfer of *Troyka* between related organisms. Horizontal transfer of transposons is much more frequent in ray-finned fishes than in mammals and birds [42]. It is very likely that horizontal transfer between closely related organisms occurs more often than between distantly related organisms. The maintenance of multiple lineages of *Troyka* in Cypriniformes is less likely, considering that no genome from Cypriniformes contains multiple active *Troyka* elements.

The lineages V1 and V2 are nested in a *Troyka* lineage from corals (Hexacorallia, Cnidaria) and the lineage V3 is in a *Troyka* lineage from scallops (Pectinidae, Bivalvia, Mollusca). These phylogenetic relationships suggest the occurrence of multiple ancient horizontal transfer events between major animal groups. The sister lineage of V3 is composed of two *Troyka* elements from *Mimachlamys varia* and *Ylistrum balloti*. Their sister lineage is constituted by *Troyka* elements from *Mizuhopecten yessoensis*, *Argopecten irradians* and *Pecten maximus*. The phylogeny of *Troyka* is not consistent with the phylogeny of host scallops; *Mimachlamys* and *Mizuhopecten* belong to the subfamily Pedinae, while *Ylistrum*, *Argopecten* and *Pecten* belong to the subfamily Pectininae. It is suggested that *Troyka* has been maintained in scallops with the combination of vertical transmission and infrequent horizontal transfer events among scallops. The lineage V3 likely originated by the horizontal transfer from scallops to fish.

The phylogeny of *Troyka* indicates that they are maintained mainly through vertical inheritance but relatively frequent horizontal transfer among related organisms and rare cross-phylum horizontal transfer cannot be negligible. The exclusive presence of *Troyka* in the aquatic environment might contribute to this pattern of maintenance. The relative stability of extracellular DNA in aquatic than terrestrial environments could be a positive factor for horizontal transfer.

### Terminal signatures and TSDs

**Fig. 2.**
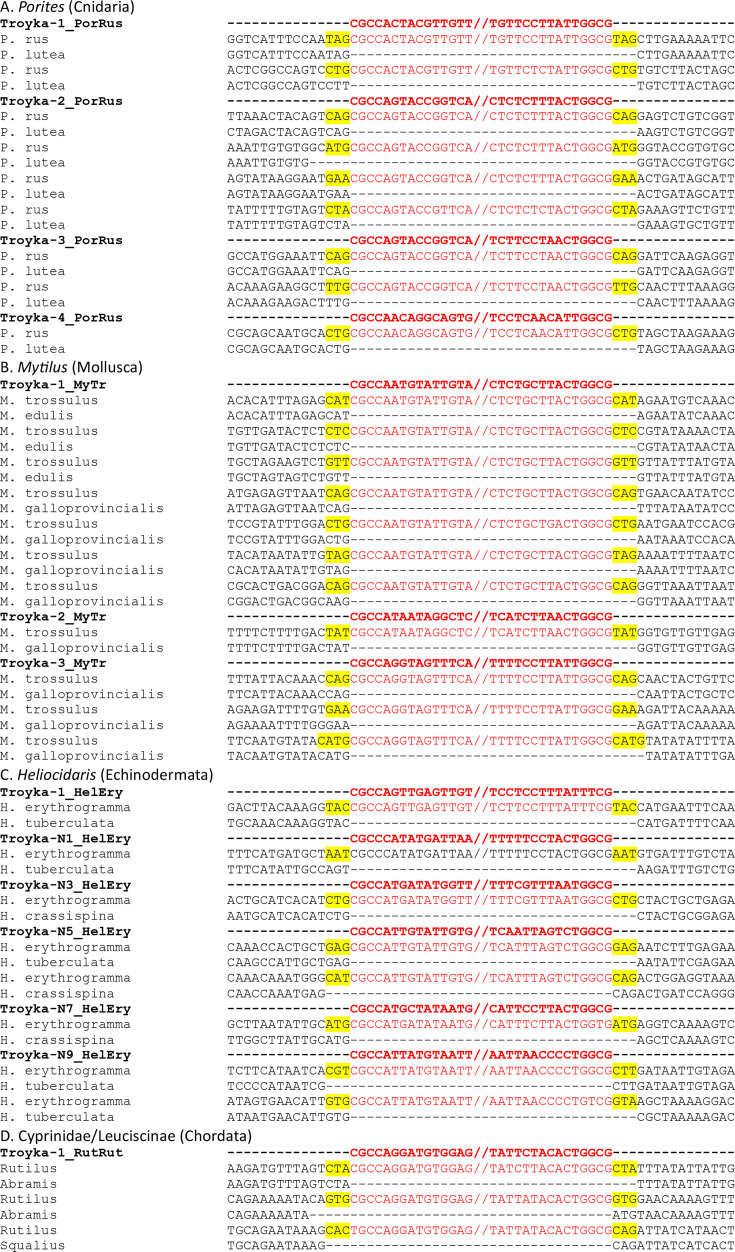
Sequence comparison between the occupied and the unoccupied sites of *Troyka* LTR retrotransposons. The consensus sequences of *Troyka* LTR retrotransposons are shown in red and bold. The *Troyka* insertions are in red. TSDs are highlighted in yellow. (**a**) *Troyka* insertions in *P. rus* and their orthologous loci in *P. lutea*. (**b**) *Troyka* insertions in *M. trossulus* and their orthologous loci in *M. edulis or M. galloprovincialis*. (**c**) *Troyka* insertions in *H. erythrogramma* and their orthologous loci in *H. tuberculata or H. crassispina*. (**d**) *Troyka* insertions in *R. rutilus* and their orthologous loci in *A. brama or S. cephalus*.

The 5′-CG.CG-3′ termini of LTRs of *Troyka* are the distinguished feature of *Troyka* in LTR retrotransposons. To confirm the termini and the alteration upon integration, the genome comparison of closely related species was performed (Fig. 2). Four groups of organisms whose genomes were sequenced were chosen for the analysis: four *Polites* species (*P. rus*, *P. lutea*, *P. cylindrica* and *P. divaricata*) in Cnidaria, five *Mytilus* species (*M. galloprovincialis*, *M. californianus*, *M. coruscus*, *M. edulis* and *M. trossulus*) in Mollusca, three *Heliocidaris* species (*H. crassispina*, *H. erythrogramma* and *H. tuberculata*) in Echinodermata and five species in Leuciscinae (*R. rutilus*, *A. brama*, *S. cephalus*, *S. erythrophthalmus* and *V. vimba*) in Chordata. The 5′-CG.CG-3′ termini and 3 bp TSDs were confirmed in all insertions whose empty loci were identified, except for one case of *Troyka-3_MyTr* in the genome of *M. trossulus*, which generated 4 bp TSDs (CATG) (Fig. 2).

Among the 603 *Troyka* LTRs characterized, 573 contain CGCCA at the 5′ terminus. The other 26 LTRs show a single transition (9 CACCA, 5 CGTCA, 4 CGCCG and 3 CGCTA) or a single transversion (2 CGCCC, 1 CGCCT, 1 CGCAA and 1 CGGCA) from CGCCA. Only four LTRs show more than two substitutions (1 CATCA, 1 CGCTG, 1 CGGCC and 1 CGGTG).

The 3′ termini of *Troyka* LTRs show more variations. Among the 603 *Troyka* LTR sequences, 522 contain TGGCG at the 3′ terminus. The 57 LTRs show a transition (18 TGACG, 4 TGGTG and 2 TGGCA) or a transversion (23 TGTCG, 8 TTGCG, 1 TGCCG and 1 TGGCT) from TGGCG. The positions −4 and −3 of TGGCG seem not to be restricted strictly, as the substitutions are found mostly (69 out of 81) only at these two positions.

These substitutions are not just mutations accumulated after transposition or errors introduced during consensus reconstruction. One *Troyka* lineage, composed of five entries (*Troyka-2_PatPel*, *Troyka-2_PatUly*, *Troyka-8_PatVul*, *Troyka-10_PatCae* and *Troyka-10_PatVul*) from the genus *Patella*, shares the TGTCG 3′ terminus. Another lineage, composed of three entries (*Troyka-3_PelNoc*, *Troyka-1_MasPap* and *Troyka-04_ClyHem*) from the two cnidarian classes (Hydrozoa and Scyphozoa) shares the TAACG 3′ terminus. One *Troyka* lineage from sea urchins includes multiple variants of 3′ terminus: 6 TTGCG, 3 TTACG, 2 TTTCG, 1 TGTCG and 1 TGGTG (Fig. S2A). One basal *Troyka* lineage from Mollusca and Annelida also shows multiple variants of the 3′ terminus: 5 TGACG and 3 TGTCG (Fig. S2B). In this lineage, two elements also show the variants at the 5′ terminus: CGTCA instead of CGCCA. These data indicate that the substitutions have been maintained during a certain evolutionary time and that the restriction of LTR termini is relaxed in these lineages.

The 5′-CG.CG-3′ termini of LTRs, along with 3 bp TSDs, are a clear indicator for the classification, even if they are not conclusive.

### Transcription

The transcription of several *Troyka* elements was confirmed by the blast search against the TSA database at NCBI (Table S2). Long transcripts that cover substantial portions of *Troyka* insertions were detected for *Troyka-1_NV* (*N. vectensis*, sea anemone), *Troyka-1_MyGa* (*M. galloprovincialis*, mussel), *Troyka-1_MyEd* (*M. edulis*, mussel), *Troyka-1_PeMa* and *Troyka-2_PeMa* (*P. maximus*, scallop) and *Troyka-2_HaRuf* (*Haliotis rufescens*, abalone) (Fig. S3). Most of these transcripts contain non-*Troyka* sequences, indicating they are read-through transcripts and are not involved in the function or transposition of *Troyka* elements. The transcript of *Troyka-2_HaRuf* (GJDU01131704.1) may represent a transcript that is used for reverse transcription, as it starts in the 5′ LTR and ends in the 3′ LTR.

### Recently active *Troyka* elements

Several *Troyka* elements seem to be active or have been active very recently. The autonomous *Troyka* elements, with an average sequence identity of greater than 99.9%, were chosen to investigate their recent activities in the respective genomes (Table S3). For each *Troyka* element, from 2 to 69 insertions were characterized with 3 bp TSDs. Recently active *Troyka* elements include *Troyka-1_AcEq* (24 insertions) from *Actinia equina*, *Troyka-1_DenCri* (25 insertions) from *Dendrophyllia cribrosa*, *Troyka-2_HelCra* (18 insertions) from *H. crassispina*, *Troyka-1_MimVar* (26 insertions) from *Mimachlamys varia* and *Troyka-1_ParEch* (43 insertions) and *Troyka-4_ParEch* (69 insertions) from *Paraescarpia echinospica*. Unfortunately, no transcription data are currently available for these *Troyka* elements, but these *Troyka* elements would be good candidates for functional characterization of this distinct lineage of retroelements.

### Phylogenetic position in virus-like retroelements

The unique characteristics of termini and target alteration let me reanalyse the phylogenetic position of *Troyka* in virus-like retroelements. *Troyka* is a type of LTR retrotransposon and was originally reported to belong to *Metaviridae*/*Gypsy* [28]. Well-studied groups of virus-like retroelements are *Metaviridae*/*Gypsy*, *Belpaoviridae*/*Bel*, *Pseudoviridae*/*Copia*, *Retroviridae*, *Caulimoviridae* and *Hepadnaviridae* [11,14]. Here, Lokiretroviruses are excluded from *Retroviridae* as they cluster with *Odin* LTR retrotransposons [21]. As *TATE*, *VIPER* and *HEART* have only a few sample sequences and they often contain mutations at conserved residues in the RT domain, these three groups were excluded from the analysis. The preliminary phylogenetic analyses indicated that *TATE* and *VIPER* are closely related to other members of *DIRS* retrotransposons and *HEART* is related to *Hepadnaviridae* and Nackednaviruses.

**Fig. 3.**
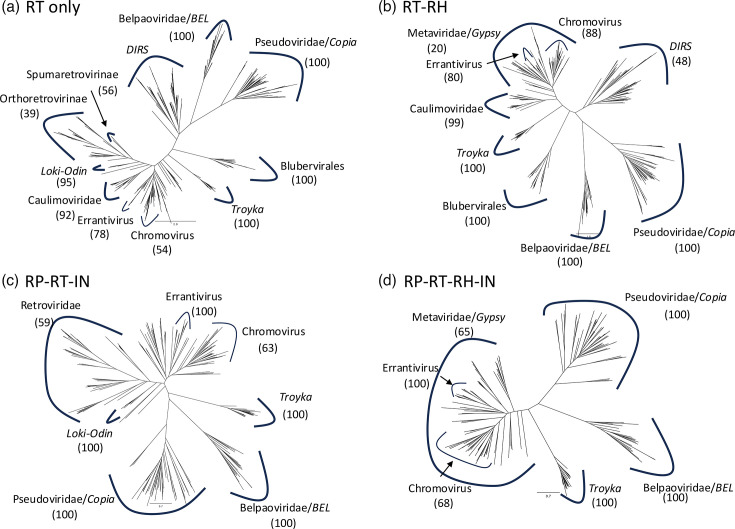
Phylogenetic trees of LTR retrotransposons and related retroelements based on the alignments of (**a**) the RT domain only; (**b**) the concatenated RT and RH domains; (**c**) the concatenated RP, RT and IN domains; and (**d**) the concatenated RP, RT, RH and IN domains. All trees are unrooted. Bootstrap values of 100 replicates for retroelement groups are shown in parentheses if available. In (**a**) and (**c**), *Metaviridae*/*Gypsy* was not indicated as a monophyletic lineage. The original phylogenetic trees and protein alignments are available as Data S4–S11.

The RH domain of *Retroviridae*, Lokiretroviruses and *Odin* LTR retrotransposons is not orthologous to those from *Troyka* [18,21, 35]. The RP domain is not found in *Hepadnaviridae*, Nackednaviruses or *DIRS*. IN domains are not found in *Caulimoviridae*, *Hepadnaviridae*, Nackednaviruses or *DIRS*. The phylogenetic trees based on the solo RT domain (RT tree) and the concatenated sequences of RT and RH domains (RT-RH tree), of RP, RT and IN (RP-RT-IN tree) and of RP, RT, RH and IN (RP-RT-RH-IN tree) were generated (Fig. 3). The monophylies of *Pseudoviridae*/*Copia*, *Belpaoviridae*/*BEL*, *Troyka*, *Caulimoviridae* and *Blubervirales* (*Hepadnaviridae* and Nackednaviruses) were well-supported. The monophyly of Lokiretroviruses and *Odin* LTR retrotransposons (*Loki-Odin*) was also supported, but the monophyly of *Retroviridae* and *Loki-Odin* was only weakly supported (35% in RT and 45% in RP-RT-IN). The monophyly of *DIRS* retrotransposons was not confirmed, even excluding *TATE* and *VIPER*.

*Metaviridae*/*Gypsy* did not constitute a well-supported distinct branch in the virus-like retroelements. The lineage of *Metaviridae*/*Gypsy* often includes the *Caulimoviridae*, *Retroviridae* and *Loki-Odin* branches. The inclusion of *Caulimoviridae* in the *Metaviridae*/*Gypsy* was also observed in previous studies [4,22], indicating that Caulimoviridae originated from *Metaviridae*/*Gypsy*. The two established internal groups of *Metaviridae*/*Gypsy*, chromoviruses and errantiviruses, were supported by over 50% bootstrap values in all trees.

**Fig. 4.**
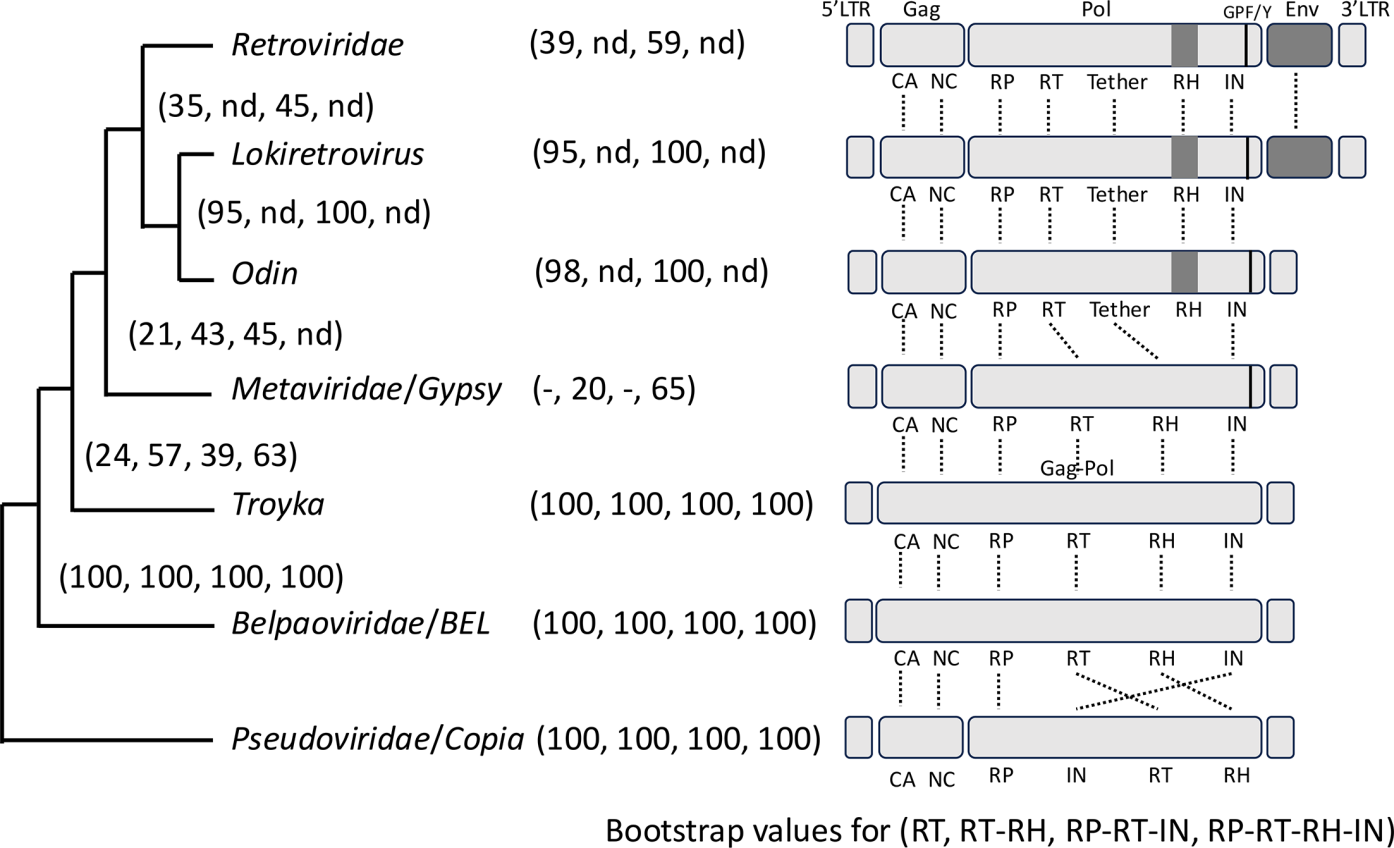
Schematic diagram of the evolution of LTR retrotransposons. Bootstrap values of 100 replicates in the phylogenetic trees of the RT only (Fig. 3a), RT-RH (Fig. 3b), RP-RT-IN (Fig. 3c) and RP-RT-RH-IN (Fig. 3d) are shown in parentheses. Minus indicates the corresponding cluster was not indicated. ‘nd’ indicates the sequences corresponding to the lineage were not included in the analysis. Canonical protein domain structures are shown at right. Vertical lines indicate the presence of the GPF/Y motif in the IN domain. CA, capsid; NC, nucleocapsid; RP, retropepsin; RT, reverse transcriptase; RH, ribonuclease H; IN, integrase.

When focusing on retroelements having LTRs, five retrotransposon groups (*Pseudoviridae*/*Copia*, *Belpaoviridae*/*BEL*, *Troyka*, *Metaviridae*/*Gypsy* and *Odin*) and two virus groups (*Retroviridae* and Lokiretroviruses) have been recognized (Fig. 4). The relationships among *Pseudoviridae*/*Copia*, *Belpaoviridae*/*BEL*, *Metaviridae/Gypsy* and *Retroviridae* have been investigated in detail [22,35, 43]. Lokiretroviruses and *Odin* LTR retrotransposons were reported as a sister lineage of *Retroviridae* [21], and the phylogenetic analysis here is consistent with the previous studies (Fig. 3).

*Troyka* is clearly distinguished from *Retroviridae*, Lokiretroviruses or *Odin* LTR retrotransposons as *Troyka* lacks the tether domain and their RH is orthologous to those from *Metaviridae*/*Gypsy* (Fig. 4). The phylogeny clearly distinguishes *Troyka* from *Pseudoviridae*/*Copia* or *Belpaoviridae*/*BEL* (Fig. 4). The remaining question is the relationship of *Troyka* with *Metaviridae*/*Gypsy*. The phylogeny of various sets of protein domain alignments generated fundamentally the same phylogenetic relationships: *Troyka* as a sister lineage of the cluster of *Metaviridae*/*Gypsy*, *Caulimoviridae*, *Retroviridae* and *Loki-Odin* (Fig. 3). There is no support for *Troyka* to be included in *Metaviridae*/*Gypsy*. The paraphyly of *Metaviridae*/*Gypsy* against *Caulimoviridae*, *Retroviridae* and *Loki-Odin* should be reinvestigated with an expanded dataset, and it would lead to the division of *Metaviridae*/*Gypsy* into several families in the virus taxonomy and superfamilies in the transposon taxonomy. The GPF/Y motif in the IN domain observed in *Metaviridae*/*Gypsy*, *Retroviridae*, *Odin* and *Ginger1* DNA transposons suggests their common ancestry [40]. The absence of the GPF/Y motif in the IN domain is another support for the position of *Troyka* outside of *Metaviridae*/*Gypsy*, although some *Metaviridae*/*Gypsy* lineages, such as chromoviruses and errantiviruses, do not have the GPF/Y motif, and thus, the lack of the GPF/Y domain could be a derived feature. Finally, the unique characteristics of termini and TSDs of *Troyka* justify their distinction from *Metaviridae*/*Gypsy*. Here, *Troyka* is proposed as a new superfamily of LTR retrotransposons in the transposon taxonomy. It could also be considered to propose a new family *Trojicaviridae* inside the order *Ortervirales* in the virus taxonomy in the future.

## Abbreviations

BIC: Bayesian information criterion
ERVs: endogenous retroviruses
IN: integrase
RH: ribonuclease H
RP: retropepsin
RT: reverse transcriptase
TERT: telomerase reverse transcriptase
TSA: transcriptome shotgun assembly
TSDs: target site duplications
